# Comparison of the Tree-Based Machine Learning Algorithms to Cox Regression in Predicting the Survival of Oral and Pharyngeal Cancers: Analyses Based on SEER Database

**DOI:** 10.3390/cancers12102802

**Published:** 2020-09-29

**Authors:** Mi Du, Dandara G. Haag, John W. Lynch, Murthy N. Mittinty

**Affiliations:** 1 School of Public Health, The University of Adelaide, 5005 Adelaide, Australia; dandara.haag@adelaide.edu.au (D.G.H.); john.lynch@adelaide.edu.au (J.W.L.); murthy.mittinty@adelaide.edu.au (M.N.M.); 2 Robinson Research Institute, The University of Adelaide, 5005 Adelaide, Australia; 3 Australian Research Centre for Population Oral Health, Adelaide Dental School, The University of Adelaide, 5005 Adelaide, Australia; 4 Population Health Sciences, University of Bristol, Bristol BS8 1QU, UK

**Keywords:** mouth neoplasms, forecasting, survivability, oropharyngeal, head and neck

## Abstract

**Simple Summary:**

Formulating accurate survival prediction models of oral and pharyngeal cancers (OPCs) is important, as they might impact the decisions of clinicians and patients. Improving the quality of these clinical prediction modelling studies can benefit the reliability of the developed models and facilitate their implementations in clinical practice. Given the growing trend on the application of machine learning methods in cancer research, we present the use of popular tree-based machine learning algorithms and compare them to the standard Cox regression as an aim to predict OPCs survival. The predictive models discussed here are based on a large cancer registry dataset incorporating various prognosis factors and different forms of bias. The comparable predictive performance between Cox and tree-based models suggested that these machine learning algorithms provide non-parametric alternatives to Cox regression and are of clinical use for estimating the survival probability of OPCs patients.

**Abstract:**

This study aims to demonstrate the use of the tree-based machine learning algorithms to predict the 3- and 5-year disease-specific survival of oral and pharyngeal cancers (OPCs) and compare their performance with the traditional Cox regression. A total of 21,154 individuals diagnosed with OPCs between 2004 and 2009 were obtained from the Surveillance, Epidemiology, and End Results (SEER) database. Three tree-based machine learning algorithms (survival tree (ST), random forest (RF) and conditional inference forest (CF)), together with a reference technique (Cox proportional hazard models (Cox)), were used to develop the survival prediction models. To handle the missing values in predictors, we applied the substantive model compatible version of the fully conditional specification imputation approach to the Cox model, whereas we used RF to impute missing data for the ST, RF and CF models. For internal validation, we used 10-fold cross-validation with 50 iterations in the model development datasets. Following this, model performance was evaluated using the C-index, integrated Brier score (IBS) and calibration curves in the test datasets. For predicting the 3-year survival of OPCs with the complete cases, the C-index in the development sets were 0.77 (0.77, 0.77), 0.70 (0.70, 0.70), 0.83 (0.83, 0.84) and 0.83 (0.83, 0.86) for Cox, ST, RF and CF, respectively. Similar results were observed in the 5-year survival prediction models, with C-index for Cox, ST, RF and CF being 0.76 (0.76, 0.76), 0.69 (0.69, 0.70), 0.83 (0.83, 0.83) and 0.85 (0.84, 0.86), respectively, in development datasets. The prediction error curves based on IBS showed a similar pattern for these models. The predictive performance remained unchanged in the analyses with imputed data. Additionally, a free web-based calculator was developed for potential clinical use. In conclusion, compared to Cox regression, ST had a lower and RF and CF had a higher predictive accuracy in predicting the 3- and 5-year OPCs survival using SEER data. The RF and CF algorithms provide non-parametric alternatives to Cox regression to be of clinical use for estimating the survival probability of OPCs patients.

## 1. Introduction

Globally, oral and pharyngeal cancers (OPCs) are ranked as the ninth most prevalent type of cancers [1]. As the only life-threatening diseases in oral health, OPCs have an estimated incidence of 834,860 new cases worldwide in 2018 [1], and have shown an increasing incidence trend over the past two decades [2]. Despite the advances in multiple types of therapies for OPCs, such as tumor removal surgery, chemo(radio)therapy and molecular-targeted therapy [3], the current 5-year overall survival rate of OPCs remains 64.8% in the United States [4]. In response to the need for improving medical care delivery in the oral health field, there are clinical decision support tools being developed to aid the early detection, diagnosis, treatment and prognosis of oral diseases, including OPCs [5]. These clinical decision support tools are all developed based on clinical prediction modelling research, which aims to yield the most accurate outcome prediction by capturing patterns in the available data (known as data-generating mechanisms) and minimizing the difference between the predicted and observed outcome (known as bias). However, following the up-to-date bias assessment criteria (PROBAST-Prediction model Risk Of Bias ASsessment Tool) [6] and reporting guidelines (TRIPOD-Transparent Reporting of a multivariable prediction model for Individual Prognosis or Diagnosis) [7], the overall quality of oral health prediction modelling studies was found to be less than optimal due to the presence of multiple sources of bias (e.g., measurement error, unmeasured predictors) and lack of reporting transparency [8].

The traditional method of survival prediction for OPCs has been building nomograms using Cox proportional hazard (Cox) regression analysis based on available clinical and sociodemographic predictors [9,10,11]. Such models are generally based on the assumptions that each predictor is linearly associated with OPCs survival outcomes. Thus, there is a possibility that these models may oversimplify complex relationships, which potentially include both non-linear associations, non-linear interactions and effect modification [12,13]. To overcome this limitation, the evolution of machine learning provides an alternative to (semi)parametric modelling by relaxing the hypothesis of the data-generating mechanism and considering all possible interactions and effect modification between variables [14]. Among the commonly used machine learning algorithms, tree-based methods (e.g., decision tree, random forest) are well-known for the ease of use, interpretability and the nature of preventing overfitting [15].

To date, despite machine learning algorithms being used for predicting OPCs prognosis [16,17], they rarely accommodate the potential systematic bias arising from data collection (e.g., missing data, measurement error) and modelling process (e.g., unmeasured predictors). Moreover, very few outputs from prediction modelling research have been implemented to assist clinical practice. Therefore, this study was performed to contribute to the clinical decision support system in the field of OPCs by (1) developing and validating various models to predict the 3- and 5-year disease-specific survival of OPCs; (2) comparing the predictive accuracy of the tree-based machine learning algorithms and the standard parametric Cox method; and (3) developing a web-based calculator to estimate the individual survival probability of OPCs patients. Additionally, this study demonstrated the conduct and reporting of a clinical prediction modelling study, following up-to-date guidelines (PROBAST and TRIPOD). The significance of this study not only lies in the development of prediction models and an online calculator for OPCs survival, but also includes a call for action to improve the quality (reduce bias) of prediction modelling studies in the field of oral health. Specifically, this study demonstrates how biases due to missing data and unmeasured predictors can be incorporated into predictive modelling.

## 2. Results

### 2.1. Patient Selection

**Figure 1:**
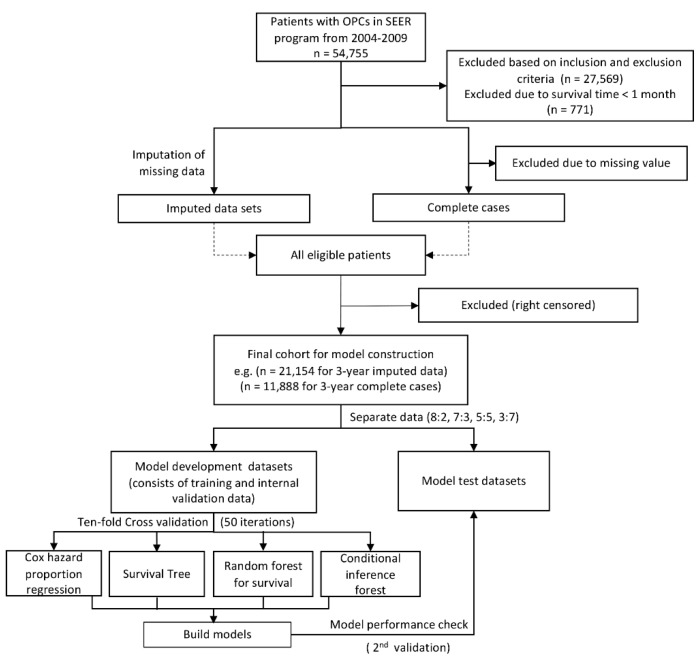
Flowchart of study design and patient selection.

A total of 54,955 primary OPC patients diagnosed between 2004 and 2009 were collected from the Surveillance, Epidemiology, and End Results (SEER) database; 27,569 records were excluded based on inclusion and exclusion selection criteria. Patients with survival months of less than 1 month were excluded (*n* = 771). Left censored samples for 3-year (*n* = 157) and 5-year (*n* = 260) cohorts were also excluded. For complete case analysis, patients who had missing values (*n* = 13,455, 49.49%) on any of the examined variables, including race (*n* = 327, 1.2%), marital status (*n* = 1745, 6.42%), tumor size (*n* = 7654, 28.15%), lymph node involvement (*n* = 2131, 7.84%), tumor (T), node (N), and metastasis (M) categories (*n* = 515, 1.89%), clinical stage (*n* = 4729, 17.39%), differentiated grades (*n* = 5803, 21.35%), surgery history (*n* = 262, 0.96%) and ICD (International Classification of Disease) classification (*n* = 437, 1.61%) were excluded. Due to the different number of left censored samples for 3- and 5-year cohorts, we ended up with 21,154 patients (21,000 for 5-year survival) and 11,888 patients (11,870 for 5-year survival) for final imputation and complete case analysis, respectively. Figure 1 shows the flowchart of patient selection.

### 2.2. Characteristics of the Patients

Here we only describe the characteristics of patients without missing information in Table 1 and Table 2. Detailed characteristics of patients in the imputed datasets can be found in Tables S1 and S2. Of the 11,888 (11,807 for 5-year survival) patients, the overall disease-specific survival rates for all patients in the 3- and 5-year cohorts were 65.0% and 60.1%, respectively. Mean survival times were 66.7 (SD = 44.1) and 66.8 (SD = 44.3) months for 3- and 5-year cohorts.

In the 3-year cohort, patients had a mean age of 59.0 (SD = 12.3) years. Of the 11,888 eligible patients, 8774 (73.8%) were male, 10,020 (84.3%) were white and 6997 (58.9%) were married at diagnosis. Overall, 7293 (61.3%) tumors arose from the oral cavity (C00-06) and tumors were poorly differentiated or undifferentiated. T3-T4 tumors accounted for 31.1% of all tumors and positive neck lymph nodes and distant metastases accounted for 44.8% and 2.9%, respectively; 7189 (60.5%) patients underwent surgery.

The 5-year cohort consisted of 11,807 patients, of which 73.9% were male, 84.3% were white and 58.9% were married at diagnosis. Similar to the 3-year cohort, over half of the tumors (61.2%) arose from the oral cavity, while 39.6% tumors were poorly differentiated or undifferentiated. T3-T4 tumors accounted for 31.2% of all tumors, lymph nodes were removed in 44.8% cases and distant metastases occurred in 2.9% tumors; 60.4% patients received surgery.

### 2.3. Model Specification

In this study, a commonly used Cox regression was chosen as the reference model and three tree-based machine learning algorithms (survival tree (ST), random forest (RF) for survival and conditional inference forest (CF)) were used to develop prediction models. These tree-based models were applied because our dataset was a mix of continuous and categorical variables, among which a large proportion had polychotomous values (i.e., more than two levels), and a major advantage of tree-based models is that they can handle this type of data by allowing for multiple splits of a selected node. Additionally, tree-based models can rank the importance of variables based on their location depth in the tree structure whereas other popular machine learning algorithms (e.g., neural networks) focus on outcome prediction with less consideration of the variables’ contribution. Here we describe the prediction models for 3-year OPC survival for the complete cases. According to the hazard ratios returned by Cox regression, all the included predictors were identified as having prognostic value for predicting OPC survival (majority of the hazard ratios with 95% confidence intervals not crossing 1) (Table S3). The two most important predictors that determine the 3- and 5-year survival of an individual patient were tumor site (hazard ratios for ICD being C02—tongue excluding base of tongue, C04—floor of mouth, C08—other salivary glands, were >3) and tumor metastasis (hazard ratio = 2.56). The final ST was with two parameters: the number of the minimum number of observations that must exist in a node (“*minsplit*”) = 11 and maximum length from root node to leaf node (“*maxdepth*”) = 18. The constructed RF model which returned the highest value of C-index included the following parameters: number of trees (“*ntree*”) = 1217, number of variables tested in any split (“*mtry*”) = 11, the size of random split points for each “*mtry*” candidate (“*nsplit*”) = 3 and split rule/formula (“*splitrule*”) = “logrank”.

### 2.4. Model Performance

The predictive performance of the models was measured using Harrell’s C-index (C-index) [18], integrated Brier score (IBS) [19] and calibration plots. Values of C-index for each model are presented in Table 3. In the main text, we present results from complete case analysis for the 8:2 split of the model development and test datasets. Results for the imputed datasets and any other splits can be found in Table S4 and Figures S1–S3.

**Figure 2:**
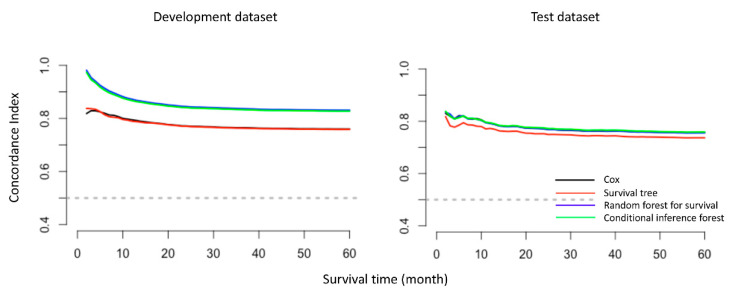
Overtime C-index for predicting disease-specific survival of oral and pharyngeal cancers with various models (Cox regression, survival tree (ST), random forest for survival (RF) and conditional inference forest (CF)) based on the complete case analysis.

For 3-year survival prediction, in the complete case analysis (*n* = 11,888), the C-indexes in development datasets were 0.77 (0.77, 0.77), 0.70 (0.70, 0.70), 0.83 (0.83, 0.84) and 0.83 (0.83, 0.86) for Cox, ST, RF and CF respectively. Similar results were found in 5-year survival cohort: CF yielded the highest values of C-index of 0.85 (0.84, 0.86), followed by RF (0.83 (0.83, 0.83)), Cox (0.76 (0.76, 0.76)) and ST (0.69 (0.69, 0.70)). The values of C-indexes in the imputed data were similar to the complete case analysis. As shown by the over-time C-index in Figure 2, RF and CF constantly exhibited the best C-index throughout the investigated period across all settings. However, this comes with a computational burden: for example, in the complete case analysis of 3-year cohort, the average training times for Cox, ST, RF and CF for each iteration were 2.78 s, 0.61 s, 348.95 s and 2513.72 s, respectively.

**Figure 3:**
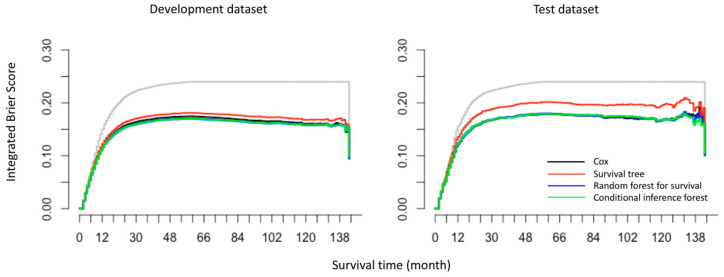
The prediction error curves for models (Cox regression, survival tree (ST), random forest for survival (RF) and conditional inference forest (CF)) in predicting disease-specific survival of oral and pharyngeal cancers based on the integrated Brier score (IBS). The maximum (observed) survival month was 143 for OPC patients in the SEER database. Different models are presented by different colors, where the grey curve represents a default benchmark Kaplan–Meier model. All curves start at time 0 where all subjects are alive and all predictions equal to 1.

**Figure 4:**
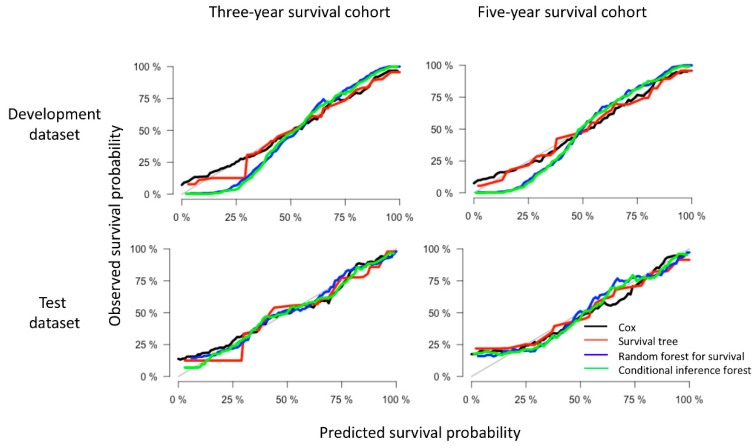
Example of calibration plots for predicting 3- and 5-year disease-specific survival of oral and pharyngeal cancers with various models (Cox regression, survival tree (ST), random forest for survival (RF) and conditional inference forest (CF)). The 45-degree straight line represents the perfect match between the observed (y-axis) and predicted (x-axis) survival probabilities. A closer distance between two curves indicates higher accuracy.

In addition to the over-time C-index plots, Figure 3 presents the prediction error curves for each model based on IBS. We found that all models performed better than the default benchmark Kaplan–Meier model. The RF and CF models (blue and green curves) had approximately the same values. Compared with the Cox model, ST showed a higher prediction error while RF and CF showed lower prediction errors in the test datasets. For the completeness and comparability of prediction models with the binary family we plotted the time-dependent receiver operator curves (ROC) based on the cumulative sensitivity and dynamic specificity for Cox models [20]. The values of area under the ROC at specific time points (1- to 5-years) were consistent with the results of over-time C-index plots. Detailed explanation and results can be found in Figure S4. In terms of calibration, the calibration curves (Figure 4) displayed by all the algorithms in the test datasets appeared close to each other, despite the weaker calibration exhibited by RF and CF in development datasets.

### 2.5. Model Presentation and Development of an Online Survival Calculator

In attempt to contribute to clinical decision-making, we have developed a web-based OPCs survival probability calculator based on a Cox regression model (this online tool was designed for research only and should not be used clinically until externally validated) (https://dumizai.shinyapps.io/apptest2/). A snapshot of the online calculator can be found in Figure S5. The potential users of this calculator are OPCs clinicians, patients and interested researchers. This calculator can (1) present the 3- and 5-year overall survival curves of OPCs cohorts in SEER database; (2) interact with the users to provide survival curves stratified by each predictor; and (3) interact with the users to provide survival probability of an individual OPCs patient for 1, 2, 3, 4 and 5 years after diagnosis.

## 3. Discussion

By comparing the prediction performance of three tree-based machine learning algorithms (ST, RF and CF) to the reference method (Cox), our findings suggest that Cox regression performed robustly as a conventional method for OPCs survival prediction; despite this, we observed an increase in C-index for RF and CF and a decrease for ST. To facilitate the translation of our developed model into clinical practice, we developed a web-based survival probability calculator to allow better visualization and ease-of-use for clinicians. It can dynamically predict the disease-specific survival probability of OPCs patients at various time points and help identify patients at high risk of OPC-specific death.

Different C-indexes obtained by Cox and tree-based models led to our further thinking on the reasons for these differences. In general, conventional statistical models (e.g., Cox) attempt to fit the data to an investigator-specified model, whereas machine learning algorithms allow the data to dictate the form of the model. In our study, Cox regression examines each predictor’s effect by testing the proportional hazards assumptions, ST focuses on data partitioning by maximizing the between-node differences, while RF and CF focus on overall prediction accuracy by reducing the prediction error. Traditional Cox regression is popular and well-studied, results are easily interpretable and it remains the most convenient solution for most survival problems. However, it is a (semi)parametric model and only works when the number of predictors is less than the number of events [21]. ST, built with “*rpart*” package [22], employed a log-rank test statistic (a statistic for comparing the survival curves of two samples) to maximize the between-subgroup heterogeneity. However, the large variance in the survival time in our study (2 to 143 months) may add complexity when distinguishing between subgroups. RF, implemented by the “*randomForestSRC*” package [23] and CF, implemented by the “*partykit*” package [19], are ensemble methods which aggregate a large number of trees. By combining thousands of trees and testing multiple node splitting rules, forest took into account all possible link functions between the outcome and predictors, as well as all possible interactions between variables. Therefore, it approximates the data-generating mechanism that is in the observed data and gains the closest predicted value to the observed value [24]. In short, in the traditional statistical sense, we selected one model (Cox), set up the model’s parameters and evaluated its accuracy. Obviously, the initial choice of algorithm would limit the flexibility of the model, while machine learning algorithms (as alternatives for prediction) have no concerns of non-proportionalities, multicollinearity or nonlinearity; thus, they may reduce the prediction bias (systematic prediction error) stemming from modelling uncertainty.

Interestingly, the predictive capabilities represented by the C-index in test datasets were similar between RF, CF and Cox for predicting OPCs survival based on the SEER database, which suggests that the superiority of machine learning is not always seen but is seen only in situations when the conventional methods meet their limits. These situations include (1) datasets with large numbers of predictors and relatively small sample size; (2) datasets with large numbers of uncorrelated candidate predictors [25] (high dimension datasets such as “omics” data); (3) datasets with complex confounding factors, interactions and nonlinearities (e.g., no clear theory or hypothesis of the proportional hazard assumption available) [26]; and (4) survival datasets with high censoring rate, where the predictors were responsible for censoring [27]. Therefore, there are several possible explanations to the comparable predictive performance between RF, CF and Cox. The first reason is the small number of predictors used in this study. Though no threshold number of predictors was available, more variables may enhance machine learning algorithms to outperform the conventional statistical models. Secondly, the predictors in the SEER program were collected based on prior clinical knowledge and many of the variables were mostly linearly correlated. Thus, there is a possibility that although RF and CF are non-parametric methods, they captured the underlying structure of data which aligned with the hypothesis that we made in the parametric model (Cox).

In summary, for prediction purposes, it is appropriate to consider a Cox model first for a given survival dataset with a smaller number of predictors. Meanwhile, machine learning algorithms can also be adopted in combination with conventional methods, so that one may obtain extra information (e.g., non-linear interactions) in the data that are not grasped by Cox models. It is also noteworthy that there is a computational burden when using RF and it cannot match the speed of the computation of Cox regression methods.

The strengths of our study include that according to PROBAST, there are a variety of systematic biases that can be present in prediction modelling studies, one of which is the bias due to missing information. In this study, we address this issue using imputed data. However, when imputing missing data, one might introduce bias by using incompatible imputation models. For example, with the imputation model being Cox regression and the final prediction model being RF, this creates incompatibility in the inference as well as estimation. We show how this can be avoided by adopting the Substantive Model Compatible Fully Conditional Specification (SMC-FCS) and RF to impute missing data for the Cox regression and tree-based models, respectively. Another bias which can occur is due to pre-specified choice of data analysis model. We address this issue by comparing both parametric and non-parametric models. We also further discuss the possible reasons that may lead to the differences of C-index between Cox and tree-based models and therefore guide users to choose different models accordingly. Finally, the web-based prediction tool for OPCs survival represents an attempt to translate research outputs into clinical practices. This prognostic tool not only informs clinicians and patients of the possible outcome of OPCs survival, but also provides suggestions to clinicians in decision-making, such as treatment determinations.

There are several limitations in this study. The main limitation of this study is the lack of records of other well-known predictors for OPCs survival in the SEER program. For example, for patients with oropharyngeal squamous cell carcinoma, individuals with human papillomavirus (HPV) positive status are likely to have better prognosis than their HPV-negative counterparts [28]. Therefore, it is worth noting that including HPV information and other prognostic factors such as smoking, alcohol consumption and chemotherapy [29] are likely to modify the models’ predictive ability. Nevertheless, sensitivity analysis was performed using the R package “*obsSens*” [30] to test the impact of unmeasured predictors on our estimates for Cox models. In the sensitivity analysis, we added another hypothetical unmeasured predictor with a different effect size (a range of 0.1–2-fold to our included predictors) on the outcome. We then examined how this added unmeasured factor impacted the estimated hazard ratios of the existing predictors. Our results showed that the conclusion might change only when the unmeasured predictor(s) had an (combined) effect on the outcome 2-fold higher than the existing predictor. This suggests that the lack of an unmeasured predictor may not inflate the effect of the existing predictors on survival outcome, and we can trust the hazard ratios and use them for new predictions. Detailed methodology and results can be found in the Supplementary Materials (Table S5). In future research, new methods are needed for incorporating sensitivity analysis for the computed estimates using the tree-based non-parametric methods. Another major limitation stemmed from the lack of validation in an external cohort; nevertheless, the replicability of the models should be sufficient with a 10-fold cross-validation method. Moreover, following the reviewer′s suggestions and also the guidance document of handling missing data in SEER [31], we presented the results from a complete case analysis in the main document. Additionally, given in the guidance document by SEER there are occasions where data can be imputed under the missing at random assumption, it is for this reason we have presented the results from imputed data in the web supplements. The results from imputed data need to be interpreted with caution. These data were imputed using the missing at random mechanism, which implies that missingness can be fully accounted for by variables that have complete information. However, this assumption might not be appropriate for all variables in the SEER data as it is linked data from multiple sources with multiple types of missingness.

Therefore, future directions derived from our study include: (1) more comprehensive models with better predictive performance can potentially be developed by adding more predictors; (2) external validation of models on another dataset (independent of the model development dataset) is required for assessing the models′ predictive capability; (3) apart from the tree-based models, multiple types of machine learning algorithms (e.g., support vector machine, neural networks) could be used for clinical prediction purposes; and (4) more research is needed to accommodate multiple sources of bias while developing a prediction model (e.g., measurement error/misclassification). We have not conducted sensitivity analysis around all the bias suggested by PROBAST, as it remains unclear how to incorporate these biases in machine learning algorithms.

## 4. Methods

### 4.1. Data Source and Study Population

Data for this study were obtained from the SEER database (approval number: 15617-Nov2017), a population-based cancer registry in the National Cancer Institute in the United States (https://seer.cancer.gov/). The University of Adelaide Human Research Ethics Committee waived the provision of ethics approval for this de-identified secondary data analysis.

The five criteria for patient inclusion were listed as follows. The first criterion was that patients were diagnosed with OPCs as the “Primary and only cancer diagnosis” during 2004 to 2009. The inception year of 2004 was chosen to allow capture of definitions of T, N and M categories as published in the sixth edition of the AJCC Cancer Staging Manual. The last year of 2009 was chosen to guarantee the completion of 5-year follow-up for each patient. The second criterion was defined by the International Classification of Disease 10th revision (ICD-10) as cancer of lips, tongue, gum, floor of mouth, palate, cheek mucosa, vestibule of the mouth and retromolar area (C00-C06), salivary glands (C07-C08), oropharynx (C09-C10), nasopharynx (C11), hypopharynx (C12-C13) [1]. The remaining three criteria were that patients had a histologically confirmed diagnosis and histological examination of squamous cell neoplasms; an active follow-up with complete dates and a known outcome with “Alive or dead due to cancer”. Patients were excluded if they had an unknown survival time, had multiple primary cancers, or were diagnosed by autopsy or death certificate (i.e., unknown date of diagnosis).

### 4.2. Predictors and Outcome

Thirteen predictor variables included in this study were age at diagnosis, sex, race, marital status at diagnosis, AJCC TNM category, overall tumor stage, histologic differentiation grade, tumor site, tumor size, whether surgical therapy was undertaken and whether lymph node was removed. All variables were categorical except age (continuous). Specifically, in SEER program, surgical therapy was recorded as “Surgery performed”, “Not recommended”, “Recommended but not performed, patient refused”, “Recommended but not performed, unknown reason”, “Recommended, but unknown performed” and “Unknown”. We have categorized this predictor as “Surgery performed”, “Surgery not performed” and “Unknown” in this study. Tumors arising from different sites (e.g., oral cavity, pharynx, salivary glands) were treated separately in our study. Detailed information on the categorization of each predictor are shown in Table 1 and Table 2. The primary outcome of interest was 3- and 5-year disease-specific survival, calculated from the date of diagnosis to the date of death due to OPCs. “Survival months” and “Death status” as outcome variables were extracted.

### 4.3. Model Development

For this study, patients were randomly assigned to a development or test dataset using a sample of 8:2 proportion, where the development dataset consisted of training and validation data. Proportion ratios of 7:3, 5:5 and 3:7 were also used to assess the models′ robustness. Cox regression, survival tree (ST), random forest (RF) and conditional inference forest (CF) were used to develop prediction models. All models were built on a development dataset using 10-fold cross-validation with 50 iterations. Samples in each iteration were randomly drawn from observed data using a different seed. After the final models were obtained, we evaluated, compared and reported the models’ predictive performance in test datasets. To explain the 10-fold cross-validation, 90% of the development data were used for training and the remaining 10% were used for validation. It was obvious that variation in the models estimates existed due to the different partitions of the data to form training and validation datasets. We adopted 10-fold cross-validation to reduce this variance by averaging over 10 different partitions, so the performance estimates were less sensitive to the random partitioning of the data. For machine learning models, all 13 predictor variables were used as inputs. The outputs were not different from the Cox regression model, which were the estimated 3- and 5-year survival probability for patients with OPCs since diagnosis. Therefore, this was a regression problem based on time-to-event (censored) data.

#### 4.3.1. Cox Regression Model

The Cox regression model can be expressed by the *hazard function* denoted by *h (t)*, which can be defined as Formula (1):

(1) $$h\left(t\right)=h_{0}\left(t\right)\;\exp\left(\alpha_{1}X_{1}+\alpha_{2}X_{2}+\ldots +\alpha_{k}X_{k}\right)$$

where t represents the survival time, $h_{0}\left(t\right)$ is the baseline hazard when all of the predictors are equal to zero. The coefficients ($\alpha_{1}$,$\;\alpha_{\;2}$,...,$\;\alpha_{k}$) measure the effect size of predictors ($X_{1},\;X_{2},\;\ldots ,\;X_{k}$).

#### 4.3.2. Survival Tree Model

A single ST can group observations according to their survival behavior based on their predictors. To grow a tree, at each node every candidate predictor is tried for node splitting. Within a set of predictors, the one with a split point that maximizes the survival differences between children nodes is finally chosen as the parent node. The growth of a decision tree is continued until the tree meets the stopping criteria, which refers to all terminal nodes containing only a minimal number of unique events which prevents further node splits (pre-defined by “*minsplit*”). Additionally, pruning procedures are used to reduce the size of the tree (pruned hyper parameters for ST can be found in Table S6). A step-by-step practical procedure of growing a ST is described in Table S7.

As for the splitting rule, the log-rank statistic can be applied [32]. The log-rank statistic maximizes the dissimilarity between children nodes using the following Formula (2):

(2) $$L\left(X,C\right)=\frac{\sum_{i=1}^{N}\left(d_{i,1}-Y_{i,1}\frac{d_{i}}{Y_{i}}\right)}{\sqrt{\sum_{i=1}^{N}\frac{Y_{i,1}}{Y_{i}}\left(1-\frac{Y_{i,1}}{Y_{i}}\right)\left(\frac{Y_{i}-d_{i}}{Y_{i}-1}\right)d_{i}}}$$

where *L(X, C)* is the log-rank measure of node separation, *X* is the predictor, *C* is the splitting point, *N* is the number of individuals in the parent node, *i* is the *i*th observation, *d_i_* is the number of deaths at time *t_i_* in the children nodes, *di,1* is the number of death in children node 1, therefore $d_{i}=\overset{t}{\underset{i=1}{\sum }}d_{i,t}$, for example when *t* = 2, then $d_{i}=d_{i,1}+d_{i,2}$. *Y_i_* are the individuals at risk at time *t_i_* and *Y_i,1_* is the number of individuals of *Y_i_* in children node 1, therefore $Y_{i}=\overset{t}{\underset{i=1}{\sum }}Y_{i,t}$

#### 4.3.3. Random Forest Model

RF is a non-parametric ensemble method that introduces two forms of randomization into the tree growing process: bootstrap sampling from the data and selection of a limited number of predictors to construct the tree (known as “*mtry*”) [25]. When growing a tree, a random *B* bootstrap sample that includes two thirds of the development data (in-bag data) is used. Based on the *B* samples, a node splitting process is applied. The node splitting process works as follows. At each node, according to a splitting criterion (pre-defined by “*splitrule*”), the predictor (among all candidate predictors, pre-defined by “*mtry*”) with a split point (pre-defined by “*nsplit*”) that maximizes the survival differences between children nodes is used for node splitting. The process of tree growing iterates “*ntree*” times to obtain a forest for final prediction. For predicting the survival of a new subject (*S^new^*) at time *t* in the *m^th^* tree, it eventually falls into a terminal node. The final prediction (Formula (3)) can be obtained by calculating node level estimation and then averaging overall trees:

(3) $${\hat{S}}^{new}\left(t\right)=\frac{1}{M}\overset{M}{\underset{m=1}{\sum }}S_{m}^{new}\left(t\right)$$

When tuning the forest, the remaining one third of the development data (out-of-bag data) was used to avoid overfitting and to select the models’ hyper parameters (Table S6) which returned the highest prediction accuracy. Details of developing a RF are described in Table S7.

#### 4.3.4. Random Forest Based on Conditional Inference Trees—Conditional Inference Forest (CF)

As we have stated above, the standard split criterion for a single ST is the log-rank test statistic, which favors splitting variables with many possible split points. Conditional inference trees can avoid this bias by using separate algorithms for selecting the best split-node from that of selecting the best splitting point [33]. Specifically, the optimal split-node is obtained by testing the association of all the available predictors to the time-to-event outcome using a linear rank test based on the log-rank transformation (log-rank score). Following this, a standard binary split is done for the selected node. CF is an ensemble model with multiple conditional inference trees. We applied CF because there were more polytomous predictors than dichotomous predictors (the number of polytomous and dichotomous predictors was 9 and 3, respectively) in our dataset and CF has been shown as superior in predictive performance to RF on time-to-event datasets with polytomous predictors [34].

### 4.4. Missing Data

When using multiple imputation (MI) methods, it is important that there is compatibility between the imputation model and the analysis model [35]. Therefore, we applied different MI methods according to these three prediction modelling approaches. For Cox regression, the SMC-FCS approach for MI [36] was used to impute missing data. Compared to the traditional FCS MI method (also known as multivariate imputation by chained equations), SMC-FCS can not only specify an appropriate regression method for imputing each predictor *X* (depending on the type of *X,* e.g., linear regression for continuous variables, logistic regression for binary variables), but also ensures that each missing value in a partially observed predictor *X* is imputed from a model (Formula (4)) that is compatible with the assumed substantive model [36]:

(4) $$f(X_{n}|Y,\;X_{-n},Z)\propto f(Y|X,\;Z)f(X_{n}|X_{-n},\;Z)$$

where $X_{n}$ refers to *n*th predictor with missing data, *Y* is the outcome and *X_−n_* refers to the remaining predictors other than the $X_{n}$ predictor. The types of regression methods used to impute each predictor when using SMC-FCS in this study can be found in Table S8.

For ST, RF and CF models, RF algorithm was used to impute missing data because parametric imputation methods (e.g., FCS) may bias our estimation due to model incompatibility. The underlying two-step imputation strategy of RF is as follows. Step 1: for each splitting node, missing data are replaced (“imputed”) with values drawn randomly from the non-missing in-bag data; Step 2: after splitting, the imputed data in the children nodes are reset to missing (then proceed as Step 1 until terminal nodes are reached). Therefore, RF imputation is carried out as a tree is being grown and all the missing values are imputed at the end of one iteration. To accommodate variance due to imputation, missing data were imputed for five sets in our study. We then performed modelling on each imputation and the estimates of models′ prediction performance over the five imputed datasets were combined using Rubin’s rules.

### 4.5. Model Validation and Performance Evaluation

Validations were conducted internally (in development datasets) and externally (in test datasets). The discriminative performance of prediction models was evaluated using an overall C-index [18], as well as a C-index which indicates the models’ C-index at each point of survival time. For example, for the ith patient, let’s say the event time is $T_{i}$, censoring time is $D_{i}$ and the predicted risk score from a model is $\eta_{i}$, and let ${\tilde{T}}_{i}=\min\left(T_{i},\;D_{i}\right)$ denote the censored time or the latest observed time and $\xi_{i}=I(T_{i}<D_{i})$ denote event indicator for right censoring. Then the C-index is an estimate of the probability that, in a randomly selected pair of cases (*i*, *j*), the sequence of events are successfully predicted [37]:

$$\mathrm{Concordance}\;\mathrm{probability}\;=\;pr(\eta_{i}>\;\eta_{j}|\;T_{i}<\;T_{j\;})$$

and the C-index is defined as Formula (5):

(5) $$C-\mathrm{index}\;=\;\frac{\sum_{i\neq j}\;I\;(\eta_{i}>\;\eta_{j})\;I\;({\tilde{T}}_{i\;}<\;{\tilde{T}}_{j})\;\xi_{i}}{\sum_{i\neq j}\;I\;({\tilde{T}}_{i}\;>\;{\tilde{T}}_{j})\xi_{i}}$$

A C-index of 0.5 or lower finds the model is predicting an outcome no better than random chance and a higher C-index corresponds to a model with higher prediction accuracy. Additionally, calibration plots, graphs consisting of two types of curves, a 45-degree straight line (reference line, indicating perfect calibration) and irregular curves (calibration curves for each model), were constructed to determine whether the predicted survival probability and observed survival probability were in concordance.

In addition to C-index, the integrated Brier score (IBS) [38] was also applied to assess models’ prediction performance. For binary prediction models, the Brier score is the mean squared prediction error. For models based on time-to-event data, the Brier score for a single subject is defined as: at a given time point *t*, the squared difference between observed event status (e.g., death) and a model based prediction of survival time *t*. The IBS represents a cumulative Brier score over time and is written as Formula (6):

(6) $$\mathrm{IBS}\left(t\right)=\frac{\;1}{t}\int_{0}^{t}E\left[{\left(I\left(T\;>\;t\right)-\;S\left(t|Z\right)\right)}^{2}\right]dF_{Z}\left(Z\right)$$

where *I*(*T* > *t*) ∈ {0, 1} is the individual survival status at time *t* and *S*(*t*|*Z*) is the predicted survival probabilities from the model with covariates *Z*. The IBS is also known as prediction error rate, where a value of 0.5 or higher indicates the model’s predictive performance is no better than a chance and a lower IBS corresponds to higher prediction ability.

### 4.6. Study Reporting and Software

For reporting the findings of this study, we followed the TRIPOD statement (Table S9). All data were extracted from SEER*Stat 8.3.5, statistical analyses were performed using STATA (Version 15, StataCorp LP, College Station, TX, USA) and R (Version 3.6.4, R Foundation for Statistical Computing, Vienna, Austria). R package “*smcfcs*” and “*randomForestSRC*” were used for missing data imputation. For model development, Cox, ST, RF and CF were fitted using R package “*mlr*” incorporated in packages “*survival*”, “*rpart*”, “*randomForestSRC*” and “*partykit*” respectively.

Data Availability: Data and codes used in this study are available via GitHub [39].

## 5. Conclusions

Based on a cohort from the SEER database, various models were used for predicting 3- and 5-year OPCs survival, where RF and CF had a higher and ST had a lower predictive capability than the reference approach (Cox regression). Moreover, a web-based calculator was developed to predict the OPCs survival probability to potentially assist clinical decision-making.

Even though no major differences in the predictive performance were seen between the imputation results and the complete case analysis, we recommend using imputation as it allows a check if there was any information loss due to missing observations. Additionally, since we are unaware of the true data-generating mechanism, it is good practice to apply multiple prediction models to check if they all lead to the same answer. This not only increases the confidence in the estimates but also increases the consistency in the estimation.

## Figures and Tables

**Table 1:** Demographic characteristics of patients with oral and pharyngeal cancers in SEER (Surveillance, Epidemiology, and End Results) cohorts.

Characteristics	3-Year Cohort (Complete Cases, *n* = 11,888)	5-Year Cohort (Complete Cases, *n* = 11,807)
**Death Status**		
Alive	7731 (65.0%)	7092 (60.1%)
Dead	4157 (35.0%)	4715 (39.9%)
**Survival months**		
Mean (SD)	66.7 (44.1)	66.8 (44.3)
Median [min, max]	77.0 [2.00, 143]	78.0 [2.00, 143]
**Age(Years)**		
Mean (SD)	59.0 (12.3)	59.0 (12.3)
Median [min, max]	58.0 [18.0, 103]	58.0 [18.0, 103]
**Sex**		
Female	3114 (26.2%)	3086 (26.1%)
Male	8774 (73.8%)	8721 (73.9%)
**Race**		
American Indian/Alaska native	55 (0.5%)	54 (0.5%)
Asian or Pacific Islander	762 (6.4%)	750 (6.4%)
Black	1051 (8.8%)	1046 (8.9%)
White	10,020 (84.3%)	9957 (84.3%)
**Marital Status**		
Divorced	1532 (12.9%)	1526 (12.9%)
Married (including common law)	6997 (58.9%)	6951 (58.9%)
Separated	135 (1.1%)	133 (1.1%)
Single (never married)	2218 (18.7%)	2198 (18.6%)
Widowed	1006 (8.5%)	999 (8.5%)

**Table 2:** Tumor-related characteristics of patients with oral and pharyngeal cancers in SEER cohorts.

Characteristics	3-Year Cohort (Complete Cases, *n* = 11,888)	5-Year Cohort (Complete Cases, *n* = 11,807)
**Differentiation Grade**		
**Well differentiated; grade I**	1535 (12.9%)	1521 (12.9%)
**Moderately differentiated; grade II**	5667 (47.7%)	5626 (47.6%)
**Poorly differentiated; grade III**	4434 (37.3%)	4410 (37.4%)
**Undifferentiated; anaplastic; grade IV**	252 (2.1%)	250 (2.1%)
**T Category**		
**T1**	3956 (33.3%)	3917 (33.2%)
**T2**	4204 (35.4%)	4171 (35.3%)
**T3**	1562 (13.1%)	1556 (13.2%)
**T4**	2132 (17.9%)	2129 (18.0%)
**TX**	34 (0.3%)	34 (0.3%)
**N Category**		
**N0**	4659 (39.2%)	4615 (39.1%)
**N1**	2416 (20.3%)	2404 (20.4%)
**N2**	4386 (36.9%)	4362 (36.9%)
**N3**	391 (3.3%)	390 (3.3%)
**NX**	36 (0.3%)	36 (0.3%)
**M Category**		
**M0**	11,447 (96.3%)	11367 (96.3%)
**M1**	348 (2.9%)	347 (2.9%)
**MX**	93 (0.8%)	93 (0.8%)
**Stage**		
**I**	2183 (18.4%)	2160 (18.3%)
**II**	1540 (13.0%)	1522 (12.9%)
**III**	2356 (19.8%)	2341 (19.8%)
**IV**	5809 (48.9%)	5784 (49.0%)
**Lymph Nodes Removed**		
**None**	6557 (55.2%)	6515 (55.2%)
**Yes**	5331 (44.8%)	5292 (44.8%)
**Tumor Size**		
**0~1 cm**	1420 (11.9%)	1404 (11.9%)
**1~2 cm**	2806 (23.6%)	2783 (23.6%)
**2~3 cm**	3120 (26.2%)	3104 (26.3%)
**3~4 cm**	2097 (17.6%)	2081 (17.6%)
**4~5 cm**	1369 (11.5%)	1363 (11.5%)
**5~6 cm**	561 (4.7%)	559 (4.7%)
**6~7 cm**	253 (2.1%)	252 (2.1%)
**7~8 cm**	128 (1.1%)	127 (1.1%)
**8~9 cm**	55 (0.5%)	55 (0.5%)
**9~10 cm**	41 (0.3%)	41 (0.3%)
**>10 cm**	38 (0.3%)	38 (0.3%)
**Surgical Therapy**		
**Surgery not performed**	4699 (39.5%)	4673 (39.6%)
**Surgery performed**	7189 (60.5%)	7134 (60.4%)
**Tumor Sites (ICD Code)**		
**Lip (C00)**	540 (4.5%)	536 (4.5%)
**Base of tongue (C01)**	2192 (18.4%)	2177 (18.4%)
**Other parts of tongue (C02)**	2400 (20.2%)	2378 (20.1%)
**Gum (C03)**	412 (3.5%)	410 (3.5%)
**Floor of mouth (C04)**	786 (6.6%)	783 (6.6%)
**Palate (C05)**	318 (2.7%)	314 (2.7%)
**Other oral cavity (C06)**	645 (5.4%)	639 (5.4%)
**Parotid gland (C07)**	253 (2.1%)	253 (2.1%)
**Other salivary glands (C08)**	39 (0.3%)	38 (0.3%)
**Tonsil (C09)**	2858 (24.0%)	2840 (24.1%)
**Oropharynx (C10)**	363 (3.1%)	362 (3.1%)
**Nasopharynx (C11)**	408 (3.4%)	404 (3.4%)
**Pyriform sinus (C12)**	391 (3.3%)	391 (3.3%)
**Hypopharynx (C13)**	283 (2.4%)	282 (2.4%)

**Table 3:** C-index in the development and test datasets for various models for predicting 3- and 5-year disease-specific survival of oral and pharyngeal cancers based on the complete case analysis.

Modelling Approaches	Development Dataset (80%) (Median (IQR))	Test Dataset (20%) (Median (IQR))
Three-Year Survival Cohort		
Cox	0.77 (0.77, 0.77)	0.76 (0.76, 0.77)
Survival tree (ST)	0.70 (0.70, 0.70)	0.70 (0.69, 0.71)
Random forest for survival (RF)	0.83 (0.83, 0.84)	0.77 (0.76, 0.77)
Conditional inference forest (CF)	0.83 (0.83, 0.86)	0.76 (0.75, 0.76)
Five-Year Survival Cohort		
Cox	0.76 (0.76, 0.76)	0.76 (0.76, 0.76)
Survival tree (ST)	0.69 (0.69, 0.70)	0.69 (0.68, 0.70)
Random forest for survival (RF)	0.83 (0.83, 0.83)	0.76 (0.76, 0.76)
Conditional inference forest (CF)	0.85 (0.84, 0.86)	0.75 (0.75, 0.76)

## Supplementary Materials

The following are available online at https://www.mdpi.com/2072-6694/12/10/2802/s1, Figure S1: Overtime C-index for predicting 3- and 5-year disease-specific survival of oral and pharyngeal cancers with various models in the imputed datasets, Figure S2: The prediction error curves for various models in predicting disease-specific survival of oral and pharyngeal cancers based on integrated Brier score in the imputed datasets, Figure S3: Calibration plots for predicting 3- and 5-year disease-specific survival of oral and pharyngeal cancers with various models in the imputed datasets, Figure S4: Time-dependent receiver operator curves for predicting 1- to 5-year disease-specific survival of oral and pharyngeal cancers with Cox models, Figure S5: Snapshot of a web-based calculator for OPCs survival probability, Table S1: Demographic characteristics of patients with oral and pharyngeal cancers in SEER cohorts (for imputed data), Table S2: Tumor-related characteristics of patients with oral and pharyngeal cancers in SEER cohorts (for imputed data), Table S3: Hazard ratios of each predictors retuned by Cox regression, Table S4: C-indexes for models predicting the 3- and 5-year disease-specific survival of OPCs in the development and test datasets (with partition ratio of 8:2, 7:3, 5:5 and 3:7), Table S5: Sensitivity analysis to investigate the effect of unmeasured factor on the estimation of hazard ratios, Table S6: Pruning parameters for survival tree and random forests for survival, Table S7: The step-by-step practical procedure of developing a ST and RF algorithm, Table S8: Type of regression methods used to impute each variable when using Multiple Imputation of Covariates by Substantive Model (smcfcs) package, Table S9: TRIPOD checklist for study reporting.
