# Supplementary material for: Comparison of the Tree-Based Machine Learning Algorithms to Cox Regression in Predicting the Survival of Oral and Pharyngeal Cancers: Analyses Based on SEER Database
Source: Cancers (Basel). 2020 Sep 29;12(10):2802. doi: 10.3390/cancers12102802 (PMC7600270; doi:10.3390/cancers12102802)
Supplement: Supplementary file 1 [file cancers-12-02802-s001.pdf]

Supplementary Materials

Comparison of the tree-based machine learning algorithms to Cox regression in predicting the survival of oral and pharyngeal cancers: Analyses based on SEER database

Mi Du[1,2,] *, Dandara Haag[1,2,3], John Lynch[1,2,4], Murthy Mittinty[1,2]

[1] School of Public Health, The University of Adelaide, South Australia, Australia
[2] Robinson Research Institute, The University of Adelaide, South Australia, Australia
[3] Australian Research Centre for Population Oral Health, Adelaide Dental School, The University of Adelaide, South Australia, Australia
[4] Population Health Sciences, University of Bristol, Bristol, United Kingdom
* Correspondence: mi.du@adelaide.edu.au; Tel.: +618 8313 3785

Table S1. Demographic characteristics of patients with oral and pharyngeal cancers in SEER cohorts (for imputed data)........- 3 -

Table S2. Tumour-related characteristics of patients with oral and pharyngeal cancers in SEER cohorts (for imputed data)........- 4 -

Table S3. Hazard ratios of each predictors returned by Cox regression. ....- 6 -

Table S4. C-indexes for models predicting the 3- and 5-year disease-specific survival of OPCs in the model development and test datasets (with partition ratio of 8:2, 7:3, 5:5 and 3:7). ....- 7 -

Figure S1. Overtime C-index for predicting 3- and 5-year disease-specific survival of oral and pharyngeal cancers with various models in the imputed datasets. ....- 9 -

Figure S2. The prediction error curves for various models in predicting disease-specific survival of oral and pharyngeal cancers based on Integrated Brier Score in the imputed datasets. ....- 9 -

Figure S3. Calibration plots for predicting 3- and 5-year disease-specific survival of oral and pharyngeal cancers with various models in the imputed datasets. ....- 9 -

Figure S4. Time-dependent receiver operator curves for predicting 1- to 5-year disease-specific survival of oral and pharyngeal cancers with Cox models.........- 10 -

Figure S5. Snapshot of a web-based calculator for OPCs survival probability. ....- 11 -

Table S5. Sensitivity analysis to investigate the effect of unmeasured factor on the estimation of hazard ratios........- 13 -

Table S6. Pruning parameters for survival tree and random forests for survival........- 14 -

Table S7. The step-by-step practical procedure of developing a ST, RF and CF algorithm. .. - 15 -

Table S8. Type of regression methods used to impute each variable when using Multiple Imputation of Covariates by Substantive Model (smcfcs) package. ....- 16 -

Table S9. TRIPOD checklist for study reporting. ....- 16 -

Table S1. Demographic characteristics of patients with oral and pharyngeal cancers in SEER cohorts (for imputed data).

	**3-YEAR COHORT**	**5-YEAR COHORT**
	Imputed data (*n*=21,154)	Imputed data (*n*=21,000)
**DEATH STATUS**		
**Alive**	13494 (63.8%)	12347 (58.8%)
**Dead**	7660 (36.2%)	8653 (41.2%)
**SURVIVAL MONTHS**		
**Mean (SD)**	65.9 (44.8)	66.0 (44.9)
**Median [min, max]**	76.0 [2.00, 143]	77.0 [2.00, 143]
**AGE(YEARS)**		
**Mean (SD)**	59.1 (12.3)	59.1 (12.3)
**Median [min, max]**	58.0 [18.0, 105]	58.0 [18.0, 105]
**SEX**		
**Female**	5451 (25.8%)	5398 (25.7%)
**Male**	15703 (74.2%)	15602 (74.3%)
**RACE**		
**American Indian/Alaska native**	112 (0.5%)	110 (0.5%)
**Asian or Pacific Islander**	1349 (6.4%)	1335 (6.4%)
**Black**	2057 (9.7%)	2044 (9.7%)
**White**	17636 (83.4%)	17511 (83.4%)
**MARITAL STATUS**		
**Divorced**	2746 (13.0%)	2701 (12.9%)
**Married (including common law)**	12188 (57.6%)	12108 (57.7%)
**Separated**	254 (1.2%)	254 (1.2%)
**Single (never married)**	4160 (19.7%)	4123 (19.6%)
**Widowed**	1806 (8.5%)	1814 (8.6%)

Table S2. Tumour-related characteristics of patients with oral and pharyngeal cancers in SEER cohorts (for imputed data).

	3-YEAR COHORT	5-YEAR COHORT
	Imputed data (*n*=21,154)	Imputed data (*n*=21,000)
**DIFFERENTIATION GRADE**		
**Well differentiated; grade I**	2911 (13.8%)	2890 (13.8%)
**Moderately differentiated; grade II**	9801 (46.3%)	9768 (46.5%)
**Poorly differentiated; grade III**	7893 (37.3%)	7805 (37.2%)
**Undifferentiated; anaplastic; grade IV**	549 (2.6%)	537 (2.6%)
**T CATEGORY**		
**T1**	5425 (25.6%)	5389 (25.7%)
**T2**	5544 (26.2%)	5500 (26.2%)
**T3**	2209 (10.4%)	2202 (10.5%)
**T4**	3659 (17.3%)	3637 (17.3%)
**TX**	4317 (20.4%)	4272 (20.3%)
**N CATEGORY**		
**N0**	7652 (36.2%)	7583 (36.1%)
**N1**	4285 (20.3%)	4255 (20.3%)
**N2**	7227 (34.2%)	7191 (34.2%)
**N3**	836 (4.0%)	831 (4.0%)
**NX**	1154 (5.5%)	1140 (5.4%)
**M CATEGORY**		
**M0**	19075 (90.2%)	18944 (90.2%)
**M1**	809 (3.8%)	806 (3.8%)
**MX**	1270 (6.0%)	1250 (6.0%)
**STAGE**		
**I**	3489 (16.5%)	3436 (16.4%)
**II**	2405 (11.4%)	2337 (11.1%)
**III**	3708 (17.5%)	3689 (17.6%)
**IV**	11552 (54.6%)	11538 (54.9%)
**LYMPH NODES REMOVED**		
**None**	13160 (62.2%)	13063 (62.2%)
**Yes**	7994 (37.8%)	7937 (37.8%)
**TUMOUR SIZE**		
**0~1cm**	2746 (13.0%)	2684 (12.8%)
**1~2cm**	4857 (23.0%)	4878 (23.2%)
**2~3cm**	5433 (25.7%)	5405 (25.7%)
**3~4cm**	3704 (17.5%)	3680 (17.5%)
**4~5cm**	2455 (11.6%)	2410 (11.5%)
**5~6cm**	1009 (4.8%)	1019 (4.9%)
**6~7cm**	463 (2.2%)	461 (2.2%)
**7~8cm**	241 (1.1%)	217 (1.0%)
**8~9cm**	100 (0.5%)	99 (0.5%)
**9~10cm**	69 (0.3%)	71 (0.3%)
**>10cm**	77 (0.4%)	76 (0.4%)
**SURGICAL THERAPY**		
**Surgery not performed**	10488 (49.6%)	10421 (49.6%)
**Surgery performed**	10666 (50.4%)	10579 (50.4%)
**TUMOUR SITES (ICD CODE)**		
**Lip (C00)**	1240 (5.9%)	1227 (5.8%)
**Base of tongue (C01)**	3989 (18.9%)	3968 (18.9%)
**Other parts of tongue (C02)**	3500 (16.5%)	3468 (16.5%)
**Gum (C03)**	612 (2.9%)	609 (2.9%)
**Floor of mouth (C04)**	1118 (5.3%)	1108 (5.3%)
**Palate(C05)**	607 (2.9%)	601 (2.9%)
**Other oral cavity(C06)**	1040 (4.9%)	1029 (4.9%)

**Parotid gland (C07)**	414 (2.0%)	411 (2.0%)
**Other salivary glands (C08)**	72 (0.3%)	70 (0.3%)
**Tonsil (C09)**	4921 (23.3%)	4885 (23.3%)
**Oropharynx (C10)**	801 (3.8%)	800 (3.8%)
**Nasopharynx (C11)**	1187 (5.6%)	1176 (5.6%)
**Pyriform sinus (C12)**	754 (3.6%)	752 (3.6%)
**Hypopharynx (C13)**	616 (2.9%)	614 (2.9%)
**Others (C14)**	283 (1.3%)	282 (1.3%)

Table S3. Hazard ratios of each predictors returned by Cox regression.

	**exp(coef)**	**exp(-coef)**	**lower .95**	**upper .95**
Age	1.0319	0.9691	1.0287	1.0351
SexMale	1.0209	0.9795	0.9430	1.1052
RaceAsian or Pacific Islander	0.6215	1.6091	0.4064	0.9503
RaceBlack	0.8914	1.1219	0.5905	1.3455
RaceWhite	0.6653	1.5030	0.4443	0.9962
Marital_sMarried (including common law)	0.7011	1.4263	0.6370	0.7717
Marital_sSeparated	0.9629	1.0386	0.7255	1.2779
Marital_sSingle (never married)	1.1360	0.8803	1.0198	1.2655
Marital_sWidowed	1.0733	0.9317	0.9409	1.2243
GradePoorly differentiated; Grade III	0.9313	1.0738	0.8663	1.0010
GradeUndifferentiated; anaplastic; Grade IV	0.9324	1.0725	0.7261	1.1972
GradeWell differentiated; Grade I	0.8087	1.2365	0.7221	0.9057
T_nT2	1.1621	0.8605	0.9507	1.4205
T_nT3	1.3497	0.7409	1.0879	1.6746
T_nT4	1.8214	0.5490	1.4871	2.2310
T_nTX	1.5970	0.6262	0.9733	2.6206
N_nN1	1.4340	0.6973	1.2673	1.6227
N_nN2	1.4997	0.6668	1.3168	1.7079
N_nN3	1.8992	0.5265	1.5593	2.3131
N_nNX	1.6248	0.6155	1.0521	2.5093
M_nM1	2.5649	0.3899	2.2253	2.9565
M_nMX	1.6576	0.6033	1.2633	2.1748
StageII	1.3670	0.7315	1.1251	1.6610
StageIII	1.2592	0.7941	1.0296	1.5400
StageIV	1.3917	0.7186	1.1263	1.7195
LN_rYes	0.8905	1.1229	0.8145	0.9737
TS_n0~1cm	0.3714	2.6924	0.2213	0.6235
TS_n1~2cm	0.5840	1.7124	0.3568	0.9559
TS_n2~3cm	0.5996	1.6679	0.3726	0.9649
TS_n3~4cm	0.7123	1.4039	0.4431	1.1451
TS_n4~5cm	0.8386	1.1924	0.5234	1.3437
TS_n5~6cm	0.8903	1.1232	0.5523	1.4352
TS_n6~7cm	0.9833	1.0170	0.6011	1.6086
TS_n7~8cm	1.2497	0.8002	0.7483	2.0870
TS_n8~9cm	1.2781	0.7824	0.7298	2.2384
TS_n9~10cm	0.8710	1.1481	0.4684	1.6198
SurgerySurgery performed	0.6187	1.6163	0.5611	0.6822
ICD_nBase of tounge (C01)	1.1606	0.8616	0.8537	1.5778
ICD_nOther parts of tounge (C02)	2.9980	0.3336	2.2311	4.0285
ICD_nGum (C03)	2.2024	0.4541	1.5841	3.0620
ICD_nFloor of mouth (C04)	3.1474	0.3177	2.3132	4.2824
ICD_nPalate(C05)	2.1562	0.4638	1.5388	3.0214
ICD_nOther oral cavity(C06)	2.9675	0.3370	2.1729	4.0526
ICD_nParotid gland (C07)	1.8092	0.5527	1.2708	2.5757
ICD_nOther salivary glands (C08)	3.1383	0.3186	1.8906	5.2093
ICD_nTonsil (C09)	0.8786	1.1381	0.6471	1.1931
ICD_nOropharynx (C10)	1.6830	0.5942	1.2041	2.3525
ICD_nNasopharynx (C11)	1.3728	0.7284	0.9671	1.9488
ICD_nPyriform sinus (C12)	2.0788	0.4810	1.5005	2.8799
ICD_nHypopharynx (C13)	2.2264	0.4492	1.5960	3.1057

Note: Results are from the complete-case analysis of training datasets (80% of the original data) of 3-year cohort.

Table S4. C-indexes for models predicting the 3- and 5-year disease-specific survival of OPCs in the model development and test datasets (with partition ratio of 8:2, 7:3, 5:5 and 3:7).

**Three-year survival cohort**		
	Development dataset (80%) (Median (IQR))	Testing dataset (20%) (Median (IQR))
Data set with complete cases (N=11,888)		
Cox	0.768 (0.767, 0.770)	0.764 (0.758, 0.768)
Survival tree	0.702 (0.701, 0.703)	0.703 (0.693, 0.705)
Random forest for survival	0.834 (0.834, 0.838)	0.766 (0.760, 0.773)
Conditional inference forest	0.833 (0.827, 0.856)	0.759 (0.755, 0.763)
Data set with imputation (N=21,154)		
Cox	0.768 (0.768, 0.769)	0.768 (0.766, 0.768)
Survival tree	0.696 (0.688, 0.706)	0.688 (0.684, 0.700)
Random forest for survival	0.831 (0.791, 0.837)	0.775 (0.771, 0.776)
Conditional inference forest	0.850 (0.838, 0.873)	0.768 (0.767, 0.770)
	Development dataset (70%) (Median (IQR))	Testing dataset (30%) (Median (IQR))
Data set with complete cases (N=11,888)		
Cox	0.769 (0.767, 0.770)	0.763 (0.760, 0.767)
Survival tree	0.702 (0.699, 0.705)	0.699 (0.693, 0.705)
Random forest for survival	0.841 (0.833, 0.841)	0.769 (0.764, 0.774)
Conditional inference forest	0.848 (0.828, 0.852)	0.775 (0.771, 0.777)
Data set with imputation (N=21,154)		
Cox	0.770 (0.769, 0.772)	0.764 (0.760, 0.765)
Survival tree	0.697 (0.690, 0.707)	0.696 (0.688, 0.701)
Random forest for survival	0.840 (0.831, 0.845)	0.776 (0.775, 0.777)
Conditional inference forest	0.843 (0.835, 0.846)	0.767 (0.765, 0.767)
	Development dataset (50%) (Median (IQR))	Testing dataset (50%) (Median (IQR))
Data set with complete cases (N=11,888)		
Cox	0.771 (0.767, 0.772)	0.764 (0.763, 0.767)
Survival tree	0.703 (0.698, 0.707)	0.696 (0.692, 0.700)
Random forest for survival	0.839 (0.828, 0.843)	0.768 (0.766, 0.769)
Conditional inference forest	0.840 (0.839, 0.842)	0.758 (0.754, 0.760)
Data set with imputation (N=21,154)		
Cox	0.771 (0.769, 0.772)	0.764 (0.763, 0.765)
Survival tree	0.698 (0.691, 0.705)	0.694 (0.686, 0.698)
Random forest for survival	0.836 (0.835, 0.842)	0.775 (0.774, 0.775)
Conditional inference forest	0.852 (0.850, 0.856)	0.771 (0.771, 0.772)
	Development dataset (30%) (Median (IQR))	Testing dataset (70%) (Median (IQR))
Data set with complete cases (N=11,888)		
Cox	0.771 (0.766, 0.774)	0.762 (0.760, 0.764)
Survival tree	0.698 (0.697, 0.710)	0.695 (0.688, 0.699)
Random forest for survival	0.842 (0.836, 0.845)	0.757 (0.762, 0.766)
Conditional inference forest	0.853 (0.851, 0.854)	0.752 (0.750, 0.756)
Data set with imputation (N=21,154)		
Cox	0.772 (0.770, 0.773)	0.765 (0.764, 0.766)
Survival tree	0.703 (0.693, 0.708)	0.689 (0.685, 0.698)
Random forest for survival	0.839 (0.830, 0.847)	0.770 (0.770, 0.772)
Conditional inference forest	0.842 (0.839, 0.850)	0.771 (0.768, 0.772)

Table continued

**Five-year survival cohort**		
	Development dataset (80%) (Median (IQR))	Testing dataset (20%) (Median (IQR))
Data set with complete cases (N=11,807)		
Cox	0.762 (0.761, 0.763)	0.756 (0.761, 0.764)
Survival tree	0.694 (0.692, 0.698)	0.688 (0.680, 0.696)
Random forest for survival	0.826 (0.826, 0.833)	0.762 (0.761, 0.764)
Conditional inference forest	0.850 (0.837, 0.856)	0.752 (0.751, 0.764)
Data set with imputation (N = 21,000)		
Cox	0.764 (0.761, 0.764)	0.762 (0.762, 0.767)
Survival tree	0.692 (0.690, 0.695)	0.689 (0.683, 0.695)
Random forest for survival	0.829 (0.828, 0.830)	0.773 (0.769, 0.776)
Conditional inference forest	0.849 (0.843, 0.854)	0.767 (0.766, 0.767)
Modelling approaches	Development dataset (70%) (Median (IQR))	Testing dataset (30%) (Median (IQR))
Data set with complete cases (N = 11,807)		
Cox	0.762 (0.761, 0.763)	0.758 (0.756, 0.761)
Survival tree	0.693 (0.692, 0.698)	0.688 (0.685, 0.694)
Random forest for survival	0.828 (0.827, 0.832)	0.760 (0.757, 0.763)
Conditional inference forest	0.843 (0.837, 0.845)	0.760 (0.759, 0.762)
Data set with imputation (N = 21,000)		
Cox	0.764 (0.761, 0.764)	0.762 (0.762, 0.767)
Survival tree	0.692 (0.690, 0.696)	0.685 (0.690, 0.695)
Random forest for survival	0.828 (0.819, 0.836)	0.772 (0.771, 0.773)
Conditional inference forest	0.850 (0.842, 0.856)	0.769 (0.766, 0.769)
	Development dataset (50%) (Median (IQR))	Testing dataset (50%) (Median (IQR))
Data set with complete cases (N = 11,807)		
Cox	0.763 (0.761, 0.765)	0.758 (0.756, 0.760)
Survival tree	0.696 (0.691, 0.701)	0.690 (0.684, 0.695)
Random forest for survival	0.825 (0.817, 0.826)	0.761 (0.759, 0.764)
Conditional inference forest	0.829 (0.821, 0.835)	0.759 (0.754, 0.761)
Data set with imputation (N = 21,000)		
Cox	0.764 (0.761, 0.764)	0.762 (0.761, 0.764)
Survival tree	0.694 (0.689, 0.700)	0.689 (0.684, 0.696)
Random forest for survival	0.824 (0.820, 0.829)	0.766 (0.766, 0.767)
Conditional inference forest	0.830 (0.830, 0.832)	0.764 (0.761, 0.765)
	Development dataset (30%) (Median (IQR))	Testing dataset (70%) (Median (IQR))
Data set with complete cases (N=11,807)		
Cox	0.764 (0.761, 0.768)	0.756 (0.754, 0.758)
Survival tree	0.696 (0.690, 0.704)	0.684 (0.681, 0.692)
Random forest for survival	0.830 (0.812, 0.835)	0.755 (0.754, 0.759)
Conditional inference forest	0.854 (0.842, 0.859)	0.756 (0.751, 0.758)
Data set with imputation (N=21,154)		
Cox	0.762 (0.762, 0.763)	0.762 (0.761, 0.762)
Survival tree	0.693 (0.690, 0.702)	0.688 (0.683, 0.692)
Random forest for survival	0.832 (0.826, 0.838)	0.764 (0.764, 0.766)
Conditional inference forest	0.850 (0.842, 0.856)	0.765 (0.759, 0.769)

Figure S1. Overtime C-index for predicting 3- and 5-year disease-specific survival of oral and pharyngeal cancers with various models in the imputed datasets.

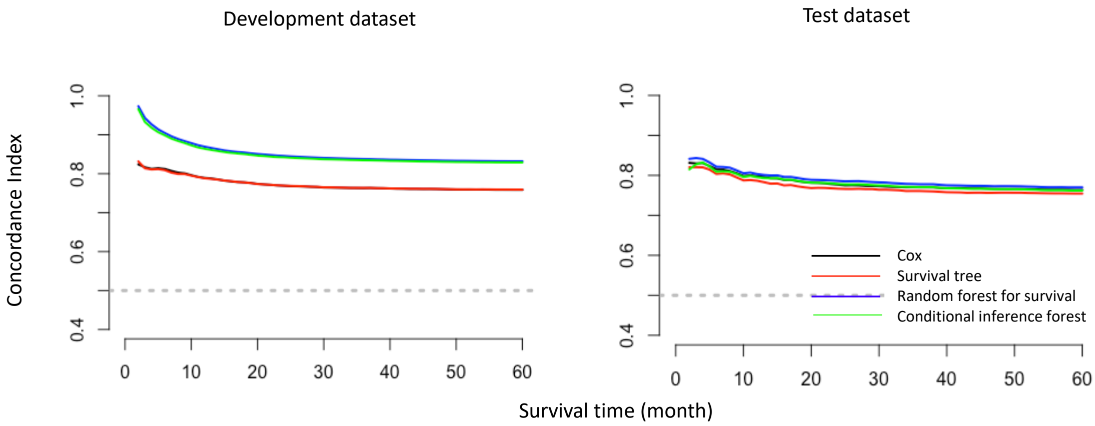

Figure S2. The prediction error curves for various models in predicting disease-specific survival of oral and pharyngeal cancers based on Integrated Brier Score in the imputed datasets.

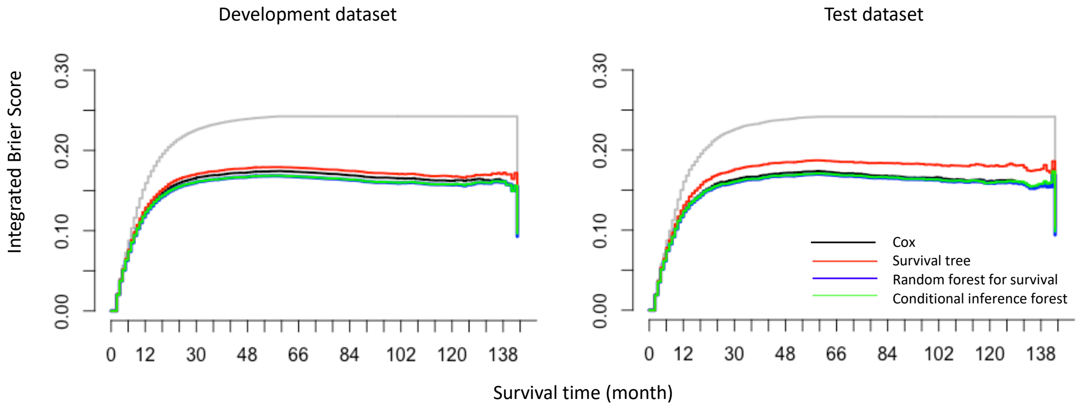

Figure S3. Calibration plots for predicting 3- and 5-year disease-specific survival of oral and pharyngeal cancers with various models in the imputed datasets.

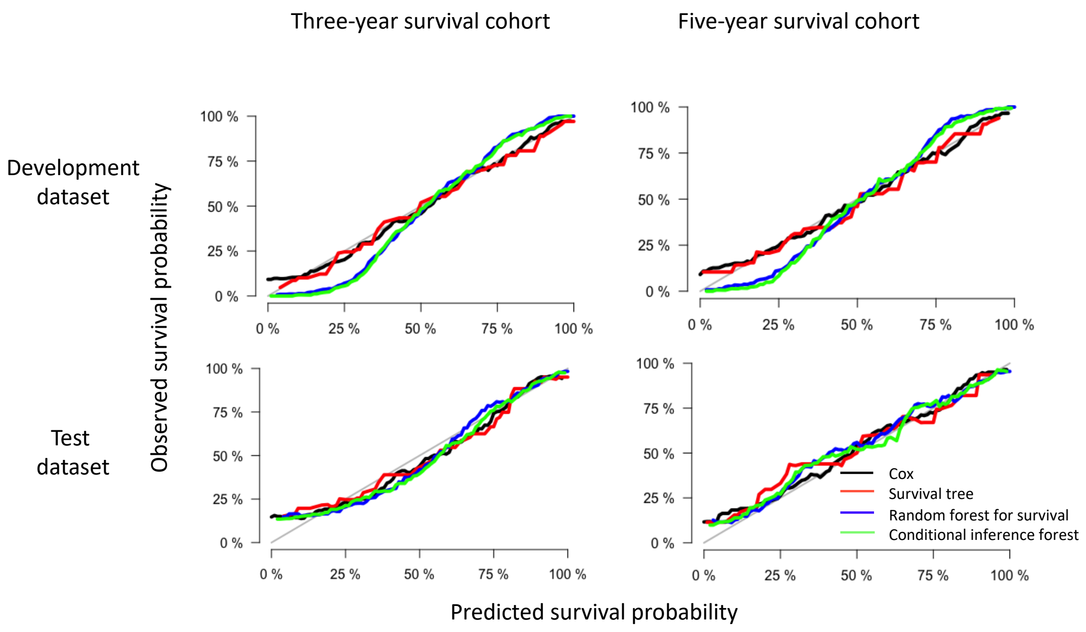

Figure S4. Time-dependent receiver operator curves for predicting 1- to 5-year disease-specific survival of oral and pharyngeal cancers with Cox models.

Description of the methods for plotting the time-dependent ROC

The time-dependent receiver operator curves (ROC) are extensions of the standard ROC curves (developed for binary data) and are developed for situations where the event status (e.g. death) occurs at various time point during the study period, and it is suitable to time-to-event analysis. The time-dependent ROC can be constructed based on the cumulative sensitivity (Se^C) and dynamic specificity (Sp^D), which have been well defined in the literature*:

Let T_i denote the predicted time of event onset and η_i is the predicted 'risk' (represented by hazard ratios of predictor values at baseline) for individual i, ($i = 1, \ldots, n$). At each observed time point t, each individual is classified as a case or control (e.g. has event/no event at that time point in between time periods $T_i = 0$ and t). A case is defined as any individual experiencing the event between baseline $t = 0$ and time t and a control as an individual remaining event-free at time t. The cases and controls change over time and each individual may play the role of control at the earlier time (when the event time is greater than the target time, i.e. $T_i > t$) but then contributes as a case for later times (when the event time is less than or equal to the target time, i.e. $T_i \leq t$). For an observed threshold c, the cumulative sensitivity of our model is defined as the probability that the individual has an predicted 'risk' greater than c among the individuals who experienced the event before time t, and the dynamic specificity is the probability that an individual has a predictor value less than or equal to c among those event-free individuals beyond time t. Thus:

$$Se^C(c,t) = P(\eta_i > c | T_i \leq t)$$

$$Sp^D(c,t) = P\ (\eta_i \leq c | T_i \geq t)$$

$$AUC^{C,D}\ (t) = P\left(\eta_i > \eta_j \middle| T_i \leq t, T_j > t\right), i \neq j$$

Results

Once the time-dependent setting is applied, the death status is observed and predicted at each time point which yields different values of sensitivity and specificity throughout the investigated time period. So that we could choose to plot the time-dependent ROC curves at a specific time of interest. For example, the following plots give the cumulative prediction performance at 12, 24, 36, 48 and 60 months.

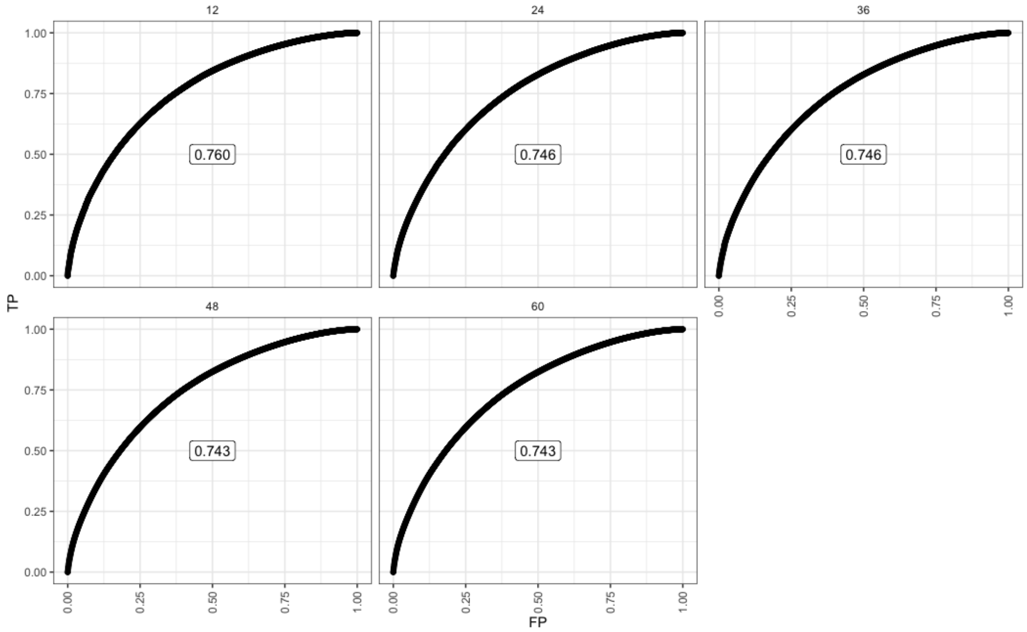

*reference: Kamarudin, Adina Najwa, Trevor Cox, and Ruwanthi Kolamunnage-Dona. Time-dependent ROC curve analysis in medical research: current methods and applications. BMC medical research methodology 17.1 (2017): 53.

Figure S5. Snapshot of a web-based calculator for OPCs survival probability.

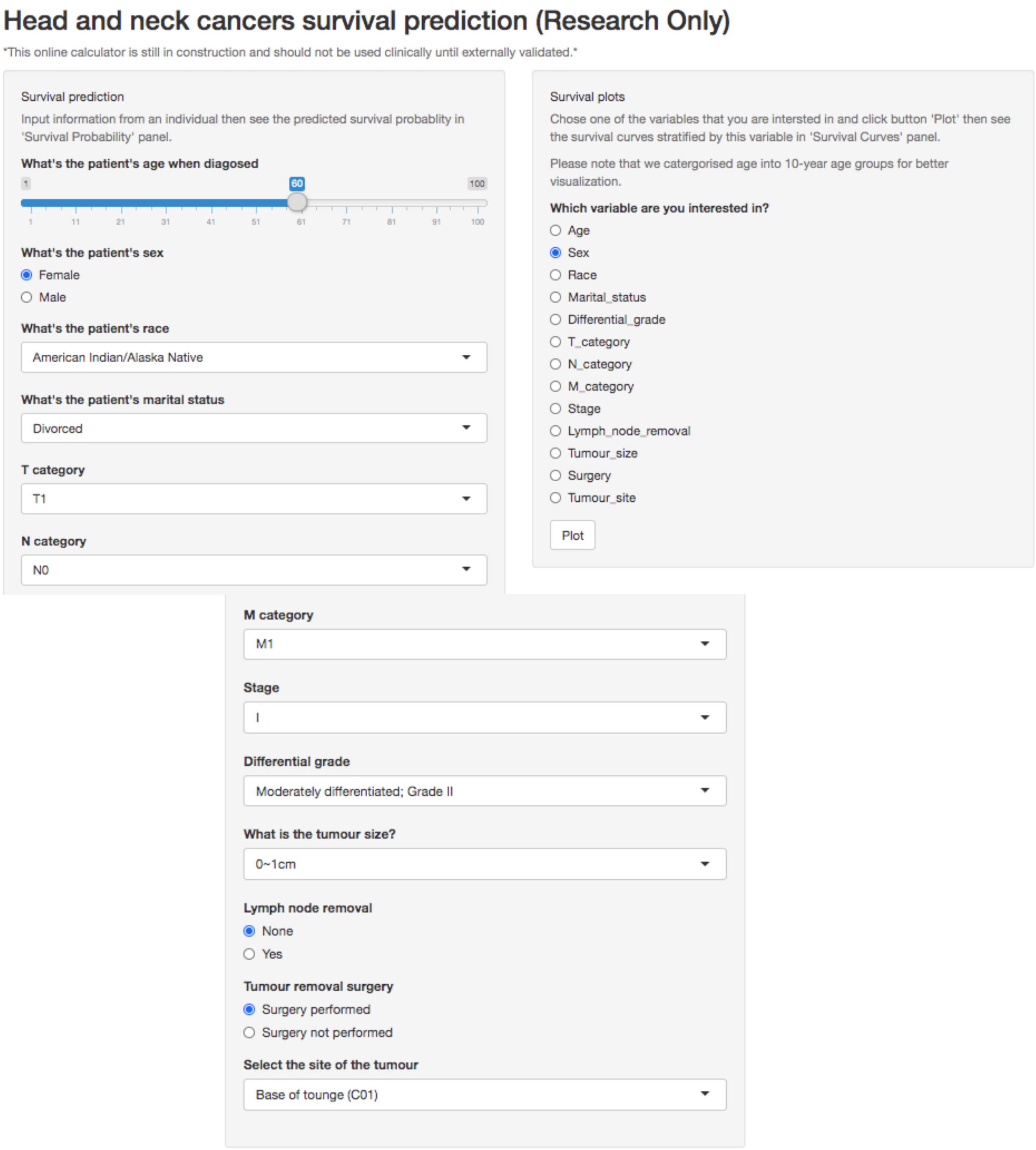
Head and neck cancers survival prediction (Research Only)
"This online calculator is still in construction and should not be used clinically until externally validated."
Survival prediction
Input information from an individual then see the predicted survival probablity in 'Survival Probability' panel.
What's the patient's age when diagosed
1
60
100
1 11 21 31 41 51 61 71 81 91 100
What's the patient's sex
Female
Male
What's the patient's race
American Indian/Alaska Native
What's the patient's marital status
Divorced
T category
T1
N category
N0
Survival plots
Chose one of the variables that you are intersted in and click button 'Plot' then see the survival curves stratified by this variable in 'Survival Curves' panel.
Please note that we catergorised age into 10-year age groups for better visualization.
Which variable are you interested in?
Age
Sex
Race
Marital_status
Differential_grade
T_category
N_category
M_category
Stage
Lymph_node_removal
Tumour_size
Surgery
Tumour_site
Plot
M category
M1
Stage
I
Differential grade
Moderately differentiated; Grade II
What is the tumour size?
0~1cm
Lymph node removal
None
Yes
Tumour removal surgery
Surgery performed
Surgery not performed
Select the site of the tumour
Base of tounge (C01)

Table S5. Sensitivity analysis to investigate the effect of unmeasured factor on the estimation of hazard ratios.

Methods

Here's an example call to the sensitivity analysis (R Code):
obsSensSCC(cox1, which=1, g0=c(0.1,0.5,2), p0=seq(0,1,0.2), p1=seq(0,1,0.2), logHaz=F)
where:

- obsSensSCC = a sensitivity analysis for three variables: outcome Y is a survival outcome, exposure X is a categorical variable such as sex, and latent variables U are categorical variables)
- model = the Cox regression model
- which = the parameter in the regression model that specifies the predictor, e.g. 2 refers to the second predictor, which was sex-male in our analysis
- g0 = strength of the relationship between U and the outcome (specified here as a hazard ratio); also called gamma
- p0 = prevalence of U in unexposed group (or when exposure = 0)
- p1 = prevalence of U in the exposed group (or when exposure = 1)
- logHaz = whether log of the hazard or the hazard ratio should be returned

In the sensitivity analysis, a range of **g** is chosen to include the unadjusted $\boldsymbol{\beta}$ before adjusting for unmeasured predictors. Together with a range of $\boldsymbol{p}$, $\boldsymbol{\beta}$ is then estimated for different values of **g** and $\boldsymbol{p}$. For example, a range of **g** of 0.1 to 2 was chosen as the hypothetical effect of unmeasured predictors that could explain away the $\boldsymbol{\beta}$ or reduce it to a specific level. If the confidence intervals of the adjusted $\boldsymbol{\beta}$ do not include 1, this suggests a direct beneficial relationship between survival outcome and predictors. On the other hand, if the confidence interval included 1, then the unmeasured predictor could explain the relationship between survival outcome and predictors.

Results

Let's look at two examples:
The following table presents the impact of unmeasured predictor on the hazard ratios of the association between two predictors and 3-year survival outcome. For the predictor 'whether the surgery was performed or not', we found that the range of $\boldsymbol{\beta}$ did not include 1 across all scenarios, which means the added unmeasured predictor did not impact on the effect of 'Surgery' on the outcome. However, for the predictor 'T category, T3', when larger proportion of T3 patients have the unmeasured predictor than the non-T3 patients (e.g. this meets the chemotherapy scenario), the effect might be changed only when the unmeasured predictors has effect 2-fold larger than the existing predictor 'T category, T3'.

```
Sensitivity analysis on variable SurgerySurgery performed
 on a Hazard Ratio scale

, , Gamma = 0.1

     P0
P1    0.2           0.4           0.6           0.8
  0.2     0.643         0.502         0.361         0.220
      (0.579,0.714) (0.452,0.558) (0.325,0.401) (0.198,0.244)
  0.4     0.824         0.643         0.462         0.281
      (0.742,0.915) (0.579,0.714) (0.416,0.513) (0.253,0.313)
  0.6     1.146         0.895         0.643         0.391
      (1.032,1.273) (0.805,0.994) (0.579,0.714) (0.352,0.435)

, , Gamma = 0.5

     P0
P1    0.2           0.4           0.6           0.8
  0.2     0.643         0.572         0.500         0.429
      (0.579,0.714) (0.515,0.635) (0.450,0.556) (0.386,0.476)
  0.4     0.723         0.643         0.563         0.482
      (0.651,0.804) (0.579,0.714) (0.507,0.625) (0.434,0.536)
  0.6     0.827         0.735         0.643         0.551
      (0.744,0.918) (0.662,0.816) (0.579,0.714) (0.496,0.612)

, , Gamma = 2

     P0
P1    0.2           0.4           0.6           0.8
  0.2     0.643         0.750         0.857         0.965
      (0.579,0.714) (0.675,0.833) (0.772,0.953) (0.868,1.072)
  0.4     0.551         0.643         0.735         0.827
      (0.496,0.612) (0.579,0.714) (0.662,0.816) (0.744,0.918)
  0.6     0.482         0.563         0.643         0.723
      (0.434,0.536) (0.507,0.625) (0.579,0.714) (0.651,0.804)
```

```
Sensitivity analysis on variable T_nT3
 on a Hazard Ratio scale

, , Gamma = 0.1

     P0
P1    0.2           0.4           0.6           0.8
  0.2     1.293         1.009         0.726         0.442
      (1.023,1.635) (0.798,1.276) (0.574,0.917) (0.349,0.558)
  0.4     1.657         1.293         0.930         0.566
      (1.311,2.095) (1.023,1.635) (0.735,1.175) (0.447,0.715)
  0.6     2.305         1.799         1.293         0.787
      (1.823,2.915) (1.423,2.275) (1.023,1.635) (0.623,0.995)

, , Gamma = 0.5

     P0
P1    0.2           0.4           0.6           0.8
  0.2     1.293         1.150         1.006         0.862
      (1.023,1.635) (0.909,1.454) (0.796,1.272) (0.682,1.090)
  0.4     1.455         1.293         1.132         0.970
      (1.151,1.840) (1.023,1.635) (0.895,1.431) (0.767,1.226)
  0.6     1.663         1.478         1.293         1.109
      (1.315,2.103) (1.169,1.869) (1.023,1.635) (0.877,1.402)

, , Gamma = 2

     P0
P1    0.2           0.4           0.6           0.8
  0.2     1.293         1.509         1.724         1.940
      (1.023,1.635) (1.193,1.908) (1.364,2.180) (1.534,2.453)
  0.4     1.109         1.293         1.478         1.663
      (0.877,1.402) (1.023,1.635) (1.169,1.869) (1.315,2.103)
  0.6     0.970         1.132         1.293         1.455
      (0.767,1.226) (0.895,1.431) (1.023,1.635) (1.151,1.840)
```

* **g(Gamma)** refers to the effect estimate of the association between predictors and an unmeasured covariate; **p** refers to the correlation between survival outcome and unmeasured covariate. Effect estimates not including 1 represent conditions where survival outcome is associated with the presence of predictors, whereas those including 1 represent conditions where survival outcome is not associated with predictors.

Table S6. Pruning parameters for survival tree and random forests for survival.

Model	Pruning Parameters
Survival tree	(‘minsplit’, lower=1,upper=20), corresponds to the minimum number of observations that must exist in a node in order for a split to be attempted.<br>(‘maxdepth’,lower=1,upper=30), corresponds to the maximum depth of a tree. Depth is the length of the longest path from a Root node to a Leaf node.
Random forests for survival	(‘ntree', lower=1000, upper=2000), corresponds to the total number of trees in the forest.<br>('mtry',lower = 1,upper = 12), corresponds to the number of variables tested in any split.<br>('nsplit',lower = 0, upper=20), corresponds to the size of random split points for each ‘mtry’ candidate.<br>('splitrule',values = 'logrank', special.vals = list('logrank','logrankscore','random')), corresponds to the split rule and formula.<br>('nodedepth',lower = …, upper =…), corresponds to the length of the longest path from a root to a leaf of any tree in the forest. The default behaviour is that this parameter is ignored.<br>('nodesize',lower =…, upper=…), corresponds to the minimum number of unique cases (data points) in a terminal node of any tree in the forest. The default behaviour is that this parameter is ignored.
Conditional Inference Forest	(‘ntree', lower=1000, upper=2000), corresponds to the total number of trees in the forest.<br>('mtry',lower = 1,upper = 12), corresponds to the number of variables tested in any split.<br>('minsplit',lower = 0, upper=20), corresponds to the minimum size of random split points for each ‘mtry’ candidate.<br>(‘teststat’, values= ‘quad’, special.vals = list(‘quad', ‘max')), corresponds to a character specifying the type of the test statistic to be applied.<br>(‘mincriterion’), corresponds to the depth of the trees. Usually unstopped and unpruned trees are used in random forests. To grow large trees, set it to a small value. The default behaviour is that this parameter is ignored.

Table S7. The step-by-step practical procedure of developing a ST, RF and CF algorithm.

The development of a ST algorithm can be summarized as follows:

1 **Start** function *F* build survival tree
2 Create an initial survival tree with root node t_0
3 Create an empty stack *S* of open nodes
4 **while** *S* is not empty **do**
5 $t = t_0 + t_1$,
if stopping criterion is met for *t* **end**
else Find the split on *F* that maximizes the survival difference between children nodes
6 Partition data in to two child nodes of *t*
7 **end**
8 **end**
9 **End**

For the tree growing and pruning purposes, one needs a splitting statistic (log-rank) that handles the dependence of failure times and a measure (C-index) to evaluate the performance of the tree.

The development of a RF algorithm can be summarized as follows:

1 **Start**
2 Select the number of trees to build, *ntree*
3 **for** *i* = 1 to *ntree* **do**
4 Generate a *B* bootstrap sample (usually two thirds) of the original data, one third is left as out-of-bag (OOB) data
5 Train a tree model on this sample
6 **for** *each split* **do**
7 Randomly select *k* (< *K*) of the original predictors
8 Select the best predictor among the *k* predictors
for *each splitting point of the best k* **do**
Compare the survival curves of the two groups using one splitting rule *r* among a) log-rank splitting rule, b) log-rank score splitting rule, or c) random log-rank splitting rule.
Select the best splitting rule and partition the data
end for determining splitting rule
9 **end** for determining *one split* in *one tree*
10 Use tree stopping criteria to determine when a tree is complete.
11 Using OOB data, the prediction ability is calculated and represented by C-index. The cumulative hazard function (CHF) is also calculated for each tree.
12 **end** for *one tree*
13 The individual C-index are then averaged to obtain the ensemble C-index. The individual CHFs are then averaged to obtain the ensemble CHF.
14 **End**

The development of a conditional inference tree can be summarized as follows:

1: For case weights *w*, test the global null hypothesis of independence between any of the p covariates and the response variable. Stop if this hypothesis cannot be rejected otherwise the the jth covariate *X* with strongest associate to the outcome.
2. Select a set $A \in X$ in order to split *X* into two disjoint sets. The weights w_L and w_R determine the two subgroups with $w_{L,i} = w_i I\ (X_{j,i} \in A)$ and $w_{R,}I = w_{iI}\ (X_{j,i} \neq A)$ for all i=1,2,…n.
3. Recursively repeat steps 1 and 2 with modeified case weights w_L and w_R, respectively.

References:

1. Ishwaran, H.; Kogalur, U.B.; Blackstone, E.H.; Lauer, M.S. Random survival forests. *Ann. Appl. Stat.* **2008**, *2*, 841–860, doi:10.1214/08-aoas169.
2. Kuhn, M. and Johnson, K., Applied predictive modeling (Vol. 26). New York: Springer. 2013.
3. Torsten Hothorn, Kurt Hornik & Achim Zeileis. Unbiased Recursive Partitioning: A Conditional Inference Framework, Journal of Computational and Graphical Statistics, 15:3, 651-674, 2006.

Table S8. Type of regression methods used to impute each variable when using Multiple Imputation of Covariates by Substantive Model (smcfcs) package.

Variables	Methods
Race, Marital status, Lymph node removal, Surgery, tumour site	'mlogit' multinomial logistic regression for unordered categorical variables
Grade, TNM category, Stage, Tumour size	'podds' proportional odds regression for ordered categorical variables
Age, Sex, Survival time, Death status	'', fully observed, does not need to be imputed

Table S9. TRIPOD checklist for study reporting.

Section/Topic	Item	Development or Validation?	Checklist Item	Page
**Title and abstract**				
Title	1	D;V	Identify the study as developing and/or validating a multivariable prediction model, the target population, and the outcome to be predicted.	1
Abstract	2	D;V	Provide a summary of objectives, study design, setting, participants, sample size, predictors, outcome, statistical analysis, results, and conclusions.	1
**Introduction**				
Background and objectives	3a	D;V	Explain the medical context (including whether diagnostic or prognostic) and rationale for developing or validating the multivariable prediction model, including references to existing models.	1-2
	3b	D;V	Specify the objectives, including whether the study describes the development or validation of the model, or both.	1-2
**Methods**				
Source of data	4a	D;V	Describe the study design or source of data (e.g., randomized trial, cohort, or registry data), separately for the development and validation datasets, if applicable.	10
	4b	D;V	Specify the key study dates, including start of accrual; end of accrual; and, if applicable, end of follow-up.	10
Participants	5a	D;V	Specify key elements of the study setting (e.g., primary care, secondary care, general population) including number and location of centres.	10
	5b	D;V	Describe eligibility criteria for participants.	10-11
	5c	D;V	Give details of treatments received, if relevant.	NA
Outcome	6a	D;V	Clearly define the outcome that is predicted by the prediction model, including how and when assessed.	11
	6b	D;V	Report any actions to blind assessment of the outcome to be predicted.	NA
Predictors	7a	D;V	Clearly define all predictors used in developing the multivariable prediction model, including how and when they were measured.	11
	7b	D;V	Report any actions to blind assessment of predictors for the outcome and other predictors.	NA
Sample size	8	D;V	Explain how the study size was arrived at.	NA
Missing data	9	D;V	Describe how missing data were handled (e.g., complete-case analysis, single imputation, multiple imputation) with details of any imputation method.	13
Statistical analysis methods	10a	D	Describe how predictors were handled in the analyses.	11
	10b	D	Specify type of model, all model-building procedures (including any predictor selection), and method for internal validation.	11-12

	10c	V	For validation, describe how the predictions were calculated.	NA
	10d	D;V	Specify all measures used to assess model performance and, if relevant, to compare multiple models.	13
	10e	V	Describe any model updating (e.g., recalibration) arising from the validation, if done.	NA
Risk groups	11	D;V	Provide details on how risk groups were created, if done.	NA
Development vs. validation	12	V	For validation, identify any differences from the development data in setting, eligibility criteria, outcome, and predictors.	NA
**Results**				
Participants	13a	D;V	Describe the flow of participants through the study, including the number of participants with and without the outcome and, if applicable, a summary of the follow-up time. A diagram may be helpful.	2-3
	13b	D;V	Describe the characteristics of the participants (basic demographics, clinical features, available predictors), including the number of participants with missing data for predictors and outcome.	3-5
	13c	V	For validation, show a comparison with the development data of the distribution of important variables (demographics, predictors, and outcome).	NA
Model development	14a	D	Specify the number of participants and outcome events in each analysis.	3-4, Table 1
	14b	D	If done, report the unadjusted association between each candidate predictor and outcome.	NA
Model specification	15a	D	Present the full prediction model to allow predictions for individuals (i.e., all regression coefficients, and model intercept or baseline survival at a given time point).	Table S3
	15b	D	Explain how to use the prediction model.	8
Model performance	16	D;V	Report performance measures (with CIs) for the prediction model.	6-8
Model updating	17	V	If done, report the results from any model updating (i.e., model specification, model performance).	NA
**Discussion**				
Limitations	18	D;V	Discuss any limitations of the study (such as nonrepresentative sample, few events per predictor, missing data).	9
Interpretations	19a	V	For validation, discuss the results with reference to performance in the development data, and any other validation data.	NA
	19b	D;V	Give an overall interpretation of the results, considering objectives, limitations, results from similar studies, and other relevant evidence.	10
Implications	20	D;V	Discuss the potential clinical use of the model and implications for future research.	8, 10
**Other information**				
Supplementary information	21	D;V	Provide information about the availability of supplementary resources, such as study protocol, Web calculator, and datasets.	14
Funding	22	D;V	Give the source of funding and the role of the funders for the present study.	14

TRIPOD: Transparent Reporting of a Multivariable Prediction Model for Individual Prognosis or Diagnosis.
